# Metabolomic Profiles of *Aspergillus oryzae* and *Bacillus amyloliquefaciens* During Rice *Koji* Fermentation

**DOI:** 10.3390/molecules21060773

**Published:** 2016-06-14

**Authors:** Da Eun Lee, Sunmin Lee, Eun Seok Jang, Hye Won Shin, Byoung Seok Moon, Choong Hwan Lee

**Affiliations:** 1 Department of Bioscience and Biotechnology, Konkuk University, Seoul 05029, Korea; daeun237@naver.com (D.E.L.); duly123@naver.com (S.L.); 2 Foods Research Institute, CJ CheilJedang Corp., Suwon 16495, Korea; eunseok.jang@cj.net (E.S.J.); hyewon.shin@cj.net (H.W.S.); bs.moon@cj.net (B.S.M.)

**Keywords:** rice *koji*, fermentation, microbe, metabolomics, enzymatic activity, antioxidant activity

## Abstract

Rice *koji*, used early in the manufacturing process for many fermented foods, produces diverse metabolites and enzymes during fermentation. Using gas chromatography time-of-flight mass spectrometry (GC-TOF-MS), ultrahigh-performance liquid chromatography linear trap quadrupole ion trap tandem mass spectrometry (UHPLC-LTQ-IT-MS/MS), and multivariate analysis we generated the metabolite profiles of rice *koji* produced by fermentation with *Aspergillus oryzae* (RK_*AO*) or *Bacillus amyloliquefaciens* (RK_*BA*) for different durations. Two principal components of the metabolomic data distinguished the rice *koji* samples according to their fermenter species and fermentation time. Several enzymes secreted by the fermenter species, including α-amylase, protease, and β-glucosidase, were assayed to identify differences in expression levels. This approach revealed that carbohydrate metabolism, serine-derived amino acids, and fatty acids were associated with rice *koji* fermentation by *A. oryzae*, whereas aromatic and branched chain amino acids, flavonoids, and lysophospholipids were more typical in rice *koji* fermentation by *B. amyloliquefaciens*. Antioxidant activity was significantly higher for RK_*BA* than for RK_*AO*, as were the abundances of flavonoids, including tricin, tricin glycosides, apigenin glycosides, and chrysoeriol glycosides. In summary, we have used MS-based metabolomics and enzyme activity assays to evaluate the effects of using different microbial species and fermentation times on the nutritional profile of rice *koji*.

## 1. Introduction

Fermented food is well-known for its nutritional benefits and biological activities [1]. Fermentation with rice *koji*, a fermented product, is widely used in the early stages of manufacturing fermented foods such as rice wine (*sake* and *makgeolli*), fermented red pepper paste (*gochujang*), and fermented soybean paste (*miso* and *doenjang*) [2,3,4]. Because rice *koji* is an enzyme source, fermentation with this product affects the quality of the fermented food. During rice *koji* fermentation, microorganisms produce enzymes involved in metabolite hydrolysis and synthesis, which enhances the flavor, taste, and bioactivities of the fermented foods [5,6]. A *nuruk* is one of the traditional starter cultures naturally fermented with various airborne microorganisms, whereas *koji* is fermented by a single microbe under regulated conditions to enhance flavor and enzymatic activity [7].

The filamentous fungus *Aspergillus oryzae*, an obligate aerobe, is the microorganism used most commonly in the production of *koji*. *A. oryzae* not only exhibit strong activity of enzymes such as amylase, protease, and peptidase, but also secretes various hydrolytic enzymes [8]. This fungus is also reported to be a safe fermenter, as under most conditions it does not produce mycotoxins [9]. In addition to *Aspergillus*, the bacterial genus *Bacillus* is widely used for fermenting soybean meal. *Bacillus* is an ideal industrial microorganism because of its high growth rate and strong capacity to produce extracellular enzymes [10]. *Bacillus subtilis* yields compounds exhibiting various biological functions, and foods fermented by *Bacillus* spp. have higher digestibility and antioxidant activity than foods fermented with *Aspergillus* spp. [11,12]. Metabolic and enzymatic differences between the fermenting species affect food qualities such as flavor, taste, and biological activity [4].

Metabolomics is considered a useful and important tool in various fields, including food science, agriculture, and microbiology. Metabolite profiling aims to monitor all metabolites in a sample, facilitating nutritional analysis [13] as well as chemotaxonomic study of plants [14] and microorganisms [15]. Metabolomic approaches have been used to investigate metabolite changes caused by filamentous fungi during rice *koji* fermentation [7]. Cooked rice fermented with different microorganisms shows different metabolite profiles, enzymatic activities, and other characteristics [2,16]; however, previous investigations have been limited to filamentous fungi and lactic acid bacteria.

Through mass spectrometry (MS)-based metabolite profiling, we identified differences between the metabolites in rice *koji* inoculated with a fungus (*A. oryzae*) and that inoculated with a bacterial species (*Bacillus amyloliquefaciens*). We also determined differences in the enzymatic and antioxidant activities of the two types of rice *koji*. Furthermore, we used these observations on metabolism to model microorganism-specific metabolic pathways.

## 2. Results

### 2.1. Multivariate Analysis of Rice Koji Fermented with Different Microorganisms and Fermentation Times

**Figure 1:**
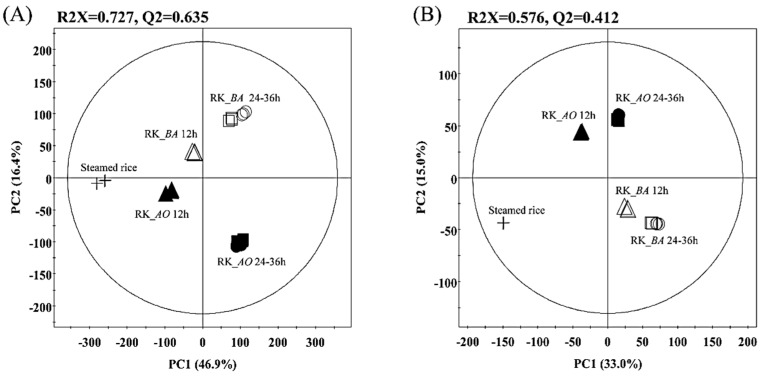
Principal component analysis (PCA) score plot for rice *koji* fermented with *A. oryzae* (RK_*AO*) or *B. amyloliquefaciens* (RK_*BA*) obtained by GC-TOF-MS (**A**) and UHPLC-LTQ-IT-MS/MS (**B**). (+, Steamed rice; unfilled symbols, RK_*AO*; filled symbols, RK_*BA*; △, ▲, 12 h; □, ■, 24 h; ○, ●, 36 h).

Metabolite profiling of the rice *koji* data sets was performed using a GC-TOF-MS and UHPLC-LTQ-IT-MS/MS combined multivariate analysis to investigate metabolite differences associated with differences in the inoculated microbes and fermentation times. The principal component analysis (PCA) score plot was obtained from GC-TOF-MS (Figure 1A) and accounted for 63.3% of the total variability. Principal Component 1 (PC1) (46.9%) distinguished the rice *koji* by fermentation time, and the rice *koji* samples fermented for 24 h and 36 h were clustered for both the *A. oryzae* fermentations (RK_*AO*) and *B. amyloliquefaciens* fermentations (RK_*BA*). The fermentation direction of RK_*AO* was clearly separated from that of RK_*BA* by Principal Component 2 (PC2) (16.4%). The partial least squares discriminant analysis (PLS-DA) showed the same pattern as the PCA analysis (supplementary meterials Figure S1). Metabolites that contributed to these distinctions in rice *koji* were identified based on their variable importance in projection values (VIP > 0.7) and *p-*values (*p* < 0.05). Forty-seven metabolites, including 10 sugars and sugar alcohols, 11 organic acids, two phenolic acids, 18 amino acids, five fatty acids, and one vitamin were determined to be important variables by PLS1 or PLS2. The metabolites were identified by comparing their mass fragment patterns and retention times with those of standard compounds, the National Institute of Standards and Technology (NIST) database (version 2.0, 2011, FairCom, Gaithersburg, MD, USA), and an in-house library. These metabolites are shown in supplementary materials Table S1, along with their relative contents represented as log_10_-transformed peak areas.

The PCA score plot acquired by UHPLC-LTQ-IT-MS/MS also presented distinct patterns associated with fermentation time (PC1: 33.0%) and inoculated microbe (PC2: 15.0%) (Figure 1B). Twenty-seven metabolites were determined by UHPLC-LTQ-IT-MS/MS to be present in significantly different (VIP > 0.7 and *p* < 0.05) quantities in rice *koji*. These metabolites included seven flavonoids, two fatty acids, nine lysophospholipids, one siderophore, and eight unknown metabolites. The metabolites were tentatively identified by comparing their molecular weights, retention times, MS^n^ fragment patterns, and UV absorbances to those in published literature and an in-house library. The relative content for each of these metabolites is shown in supplementary materials Table S2, with peak areas log_10_ transformed.

### 2.2. Different Metabolites and Metabolic Pathway of Rice Koji According to Microorganisms

**Figure 2:**
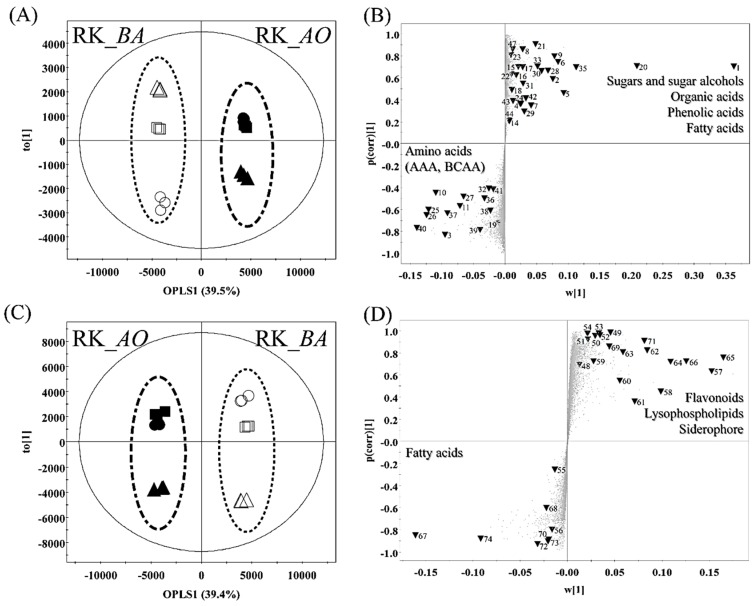
The OPLS-DA score plot (**A**,**C**) and loading S-plot (**B**,**D**) for rice *koji* fermented with *A. oryzae* (RK_*AO*) or *B. amyloliquefaciens* (RK_*BA*) obtained by GC-TOF-MS (**A**,**B**), and UHPLC-LTQ-IT-MS/MS (**C**,**D**). Highlighted metabolites (▼) in the S-plot indicate statistically significant differences between RK_*AO* and RK_*BO* (VIP > 1.0 and *p* < 0.05 in OPLS-DA). Each labeled peak number indicates a metabolite in Tables S1 and S2. The stated super-classes contain the identified metabolites (AAA, aromatic amino acid; BCAA, branched chain amino acid).

To confirm that the metabolites of rice *koji* differed according to the inoculated microorganism, RK_*AO* (12–36 h) and RK_*BA* (12–36 h) were subjected to an orthogonal partial least square discriminant analysis (OPLS-DA), which showed a clear separation by OPLS component 1, accounting for 39.5% and 39.4% of the variance in data obtained from the GC-TOF-MS (Figure 2A) and UHPLC-LTQ-IT-MS/MS analyses (Figure 2C), respectively. 

We highlight the 69 metabolites selected as variables (VIP > 1.0, *p-*value < 0.05) in the S-plots obtained from GC-TOF-MS (Figure 2B) and UHPLC-LTQ-IT-MS/MS (Figure 2D). Results showed that the levels of eight sugars and sugar alcohols, seven organic acids, two phenolic acids, seven amino acids, five fatty acids, two nucleotides, one vitamin, and six unknown compounds were higher in RK_*AO* than in RK_*BA*. Two sugars and sugar alcohols, two organic acids, 10 amino acids, seven flavonoids, nine lysophospholipids, one siderophore, and two unknown compounds were major metabolites in RK_*BA*.

**Figure 3:**
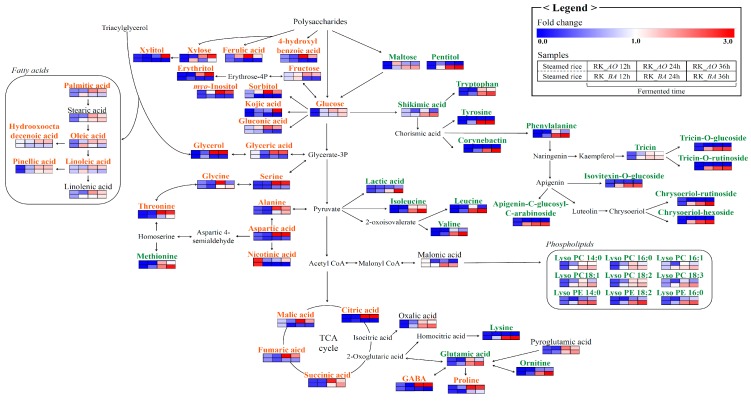
Scheme of the metabolic pathway and relative metabolite contents in rice *koji* fermented with *A. oryzae* (RK_*AO*) or *B. amyloliquefaciens* (RK_*BA*). The pathway was retrieved from the KEGG database and modified (KEGG. http://www.genome.jp/kegg). The colored squares (blue-to-red) represent fold changes normalized by the average of all values for each metabolite. The orange characterized metabolites had significantly higher relative contents in RK_*AO*, while the green characterized metabolites had higher contents in RK_*BA* (VIP > 1.0 and *p* < 0.05 in OPLS-DA).

Figure 3 depicts the metabolic pathways shared by the two microbes of interest. Beneath each metabolite, the color on a blue-to-red gradient indicates the mean-normalized relative abundance of each metabolite under each experimental condition, as determined by MS, and metabolites that were significantly different between RK_*AO* and RK_*BA* in the OPLS-DA models are shown in orange (RK_*AO*) and green (RK_*BA*). Carbohydrate metabolism-related metabolites such as sugars and sugar alcohols (glycerol, erythritol, xylose, xylitol, fructose, glucose, sorbitol, and *myo-*inositol), organic acids (succinic acid, glyceric acid, fumaric acid, malic acid, kojic acid, citric acid, and gluconic acid), and phenolic acids (4-hydroxybenzoic acid and ferulic acid) were more abundant in the RK_*AO* samples than they were in RK_*BA* samples. Related to amino acid metabolism, the aromatic amino acids (AAA; phenylalanine, tyrosine, and tryptophan) derived from shikimic acid and the branched chain amino acids (BCAA; valine, leucine, and isoleucine) were higher in RK_*BA*, while amino acids related to the serine pathway (alanine, glycine, serine, threonine, and aspartic acid) were higher in RK_*AO*. The flavonoids (apigenin-C-glucosyl-C-arabinoside, isovitexin-*O*-glucoside, chrysoeriol-hexoside, chrysoeriol-rutinoside, tricin-7-*O*-rutinoside, tricin-*O*-glucoside, and tricin) originated from shikimic acid metabolism and were relatively abundant in RK_*BA*. In the case of lipid metabolism, the relative content of fatty acids (palmitic acid, linoleic acid, oleic acid, pinellic acid, and hydroxy-oxo-octadecenoic acid) and lysophospholipids (lysoPC (lysophosphatidylcholine) 14:0, LysoPC 18:3, LysoPC 16:1, LysoPC18:2, LysoPC 16:0, LysoPC 18:1, LysoPE (lysophosphatidyl-ethanolamine) 14:0, LysoPE 18:2, and LysoPE16:0) showed opposite trends according to each microorganism.

### 2.3. Comparison of Bioactivity and Enzymatic Activity in Different Rice Koji Depending on Microorganisms

**Figure 4:**
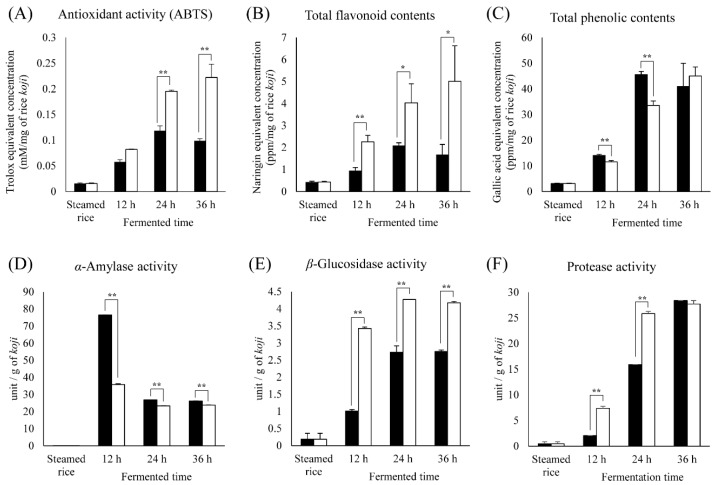
Comparison of bioactivities and enzymatic activities for rice *koji* fermented with *A. oryzae* (RK_*AO*, filled columns) or *B. amyloliquefaciens* (RK_*BA*, unfilled columns) for different durations. The bioactivities are antioxidant activity (ABTS) (**A**); total flavonoid contents (**B**); and total phenolic contents (**C**); The enzymatic activities are α-amylase activity (**D**); β-glucosidase activity (**E**); and protease activity (**F**). Significant differences between the RK_*AO* and RK_*BA* groups were identified by *t*-test (* *p* < 0.05, ** *p* < 0.01).

To further examine the differences between rice *koji* inoculated with *A. oryzae* and that inoculated with *B. amyloliquefaciens* during fermentation, we evaluated the antioxidant activity (ABTS), total flavonoid contents (TFC), and total phenolic contents (TPC) (Figure 4A–C). The overall levels of ABTS and TFC were higher for RK_*BA* than for RK_*AO*. In case of TPC, RK_*AO* had higher level than RK_*BA* at 24 h and 12 h, and there is no significant difference at 36 h. The antioxidant activity of RK_*BA* increased monotonically with fermentation time, whereas that of RK_*AO* increased until 24 h and then decreased; we observed an analogous pattern for TFC and TPC. This pattern in TFC and TPC was also almost analogous with the flavonoids and phenolic compounds detected by MS-based analysis.

To understand the differences in metabolites according to the inoculated microorganisms during fermentation, the enzymatic activities in rice *koji* were measured (Figure 4D–F). The α-amylase activity was highest in the 12-h RK_*AO* group, and all RK_*AO* groups exhibited stronger α-amylase activities than the RK_*BA* groups. β-Glucosidase and protease activities were higher in the RK_*BA* groups than in the RK_*AO* groups. Enzyme activities exhibited wider ranges at early-mid fermentation (0–24 h) phase than at later fermentation (24–36 h).

## 3. Discussion

Enzymes produced by inoculated microorganisms affect the repertoire of metabolites found in *koji* fermentations. This relationship means that microbial fermenters have decisive effects on the taste, flavor, and nutritional value of the final product [4]. Although there are many scientific studies on rice *koji* fermentation, only filamentous fungi have been used in non-targeted metabolomic approaches. We investigated differences in microbial metabolites along with antioxidant and enzymatic activities in metabolic pathways between rice *koji* prepared with two inoculated microbes, a fungus (*A. oryzae*) and a bacterium (*B. amyloliquefaciens*), to better understand the nutritional qualities of rice *koji. Bacillus* species are regarded one of the most potent microbial fermenters because of their rapid growth rate, strong secreted enzymes, and the ability enhancing bioactivity.

**Figure 5:**
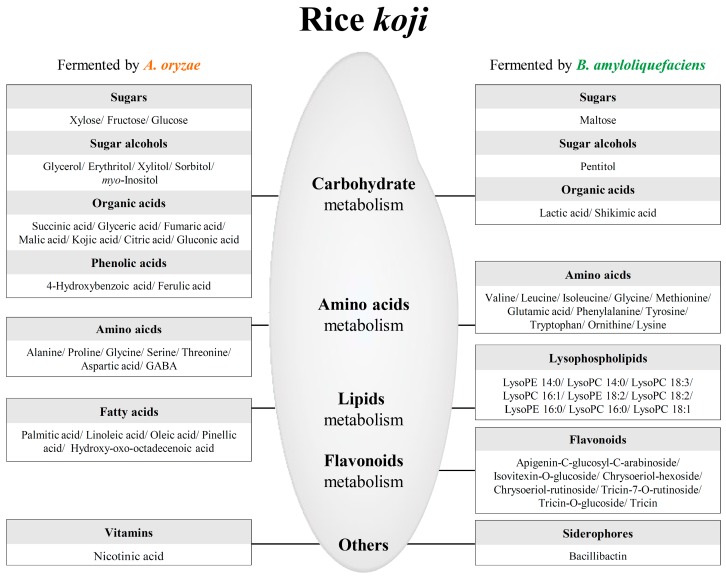
The metabolic comparison between rice *koji* fermented with *A. oryzae* for 24 h (RK_*AO* 24 h) or *B. amyloliquefaciens* for 24 h (RK_*BA* 24 h). The selected metabolites are variables of OPLS-DA in Figure 2. The metabolite located on left has higher relative content in RK_*AO* 24 h, while that of located on right was higher in RK_*BA* 24 h.

The metabolite and enzymatic activities in rice *koji* are largely distinguished by fermentation time and fermenter species (Figure 1 and Figure 4). The abundances of most metabolites increased with fermentation time and showed remarkable changes in early-mid fermentation (0–24 h), whereas there was no great difference in late fermentation (24–36 h) in both RK_*AO* and RK_*BA*. These changes in metabolites and enzymatic activities depending on fermentation time showed patterns that were similar to those found in previous studies [17,18]. This result may indicate that the early-mid fermentation (0–24 h) is the most influential stage for rice *koji*, as far as the metabolome is concerned. This comparison also revealed great metabolic differences between fermentations with each of the microorganisms. Because many enzymes, encoded by multiple genes and involved in many pathways, participate in the biosynthesis and degradation of a single metabolite, it is difficult to clearly explain the changes in metabolites observed during rice *koji* fermentation. Nevertheless, the MS-based metabolomic approach reveals that the fungus *A. oryzae* and the bacterium *B. amyloliquefaciens* have different metabolic strategies for fermentation of rice *koji*. Figure 5 showed the metabolic comparison between rice *koji* by two microbes for 24 h because metabolite difference was noticeable in this sample. Selected metabolites had statistically significant differences in OPLS-DA between RK_*AO* and RK_*BO* (VIP > 1.0, *p* < 0.05).

### 3.1. Sugars and Sugar Alcohols

Sugars are generally consumed as carbon sources, yielding energy for proliferation and growth *via* carbohydrate metabolic pathways such as glycolysis, the pentose phosphate pathway, and the tricarboxylic acid (TCA) cycle. Polysaccharides must be degraded by microbial enzymes such as α- or β-amylase or β-glycosidase before being absorbed [19]. Our results showed that β-glucosidase activity (Figure 4E) was higher in the RK_*BA* groups than in the RK_*AO* groups, whereas sugar (Figure 3), sugar alcohol (Figure 3), and α-amylase activities (Figure 4D) were higher in the RK_*AO* groups. β-Glucosidase liberates glucose by cleaving glucosidic linkages between oligosaccharides and flavonoid glycosides [20]. α-Amylase decomposes rice starch into disaccharides and oligosaccharides, which helps in the production of monosaccharides and sugar alcohols in carbohydrate microbial metabolism [21]. *A. oryzae* releases various sugar metabolism-related enzymes in addition to β-glucosidase and α-amylase. In the case of xylose metabolism, the xylitol content was far higher in RK_*AO* than in RK_*BA*. Fernandes *et al.* [22] studied pentose metabolism and reported that fungi convert xylose and arabinose to xylitol and arabitol through NADPH-consuming reactions, while bacteria do not transform xylose to xylitol. Other sugar alcohols such as sorbitol and erythritol are also converted from monosaccharides by NADPH-dependent aldose reductase, which is mainly detected in fungi [23,24,25]. The cooperation of enzymes such as amylolytic enzymes and reductases provides microbes, particularly *A. oryzae*, with the ability to vigorously metabolize carbohydrates.

### 3.2. Organic Acids

In carbohydrate metabolism, sugar reduction causes organic acid production during fermentation, influencing the acidity of the culture environment and its habitability for microbes [26]. The filamentous fungi produce various organic acids, especially citric acid and gluconic acid [27,28]. Seven organic acids, including TCA cycle intermediates, had higher relative contents in RK_*AO* than in RK_*BA* (Figure 3). RK_*AO* samples exhibited a lower pH than RK_*BA* samples, because of their heightened organic acid content (supplementary materials Figure S2). This result is consistent with a previous report that the acidity of *gochujang* made with *Bacillus* species-fermented *koji* was lower than that of *gochujang* made with *A. oryzae*-fermented *koji* [18]. *A. oryzae* tolerates acidic conditions, showing a broad acceptable pH range between 3 and 7, although low pH conditions may inhibit microbial growth [29]. Lactic acid and shikimic acid are organic acids that were predominantly detected in RK_*BA* (Figure 2). Lactic acid is produced naturally during fermentation by pyruvate metabolism (Figure 3). Lactate dehydrogenase catalyzes the reciprocal conversion of pyruvate and lactic acid; high lactate dehydrogenase activity was demonstrated in *Bacillus* species, but not in *A. oryzae* [30]. Lactic acid production by fungus is difficult because near neutral pH conditions must be maintained and the production of ethanol and fumaric acid interrupt the process [31]. In microbial metabolism, shikimic acid is produced via several steps from phosphoenolpyruvate and erythrose 4-phosphate, precursors derived from glycolysis [32]. Shikimic acid is linked to the biosynthesis of AAA such as tryptophan, tyrosine, and phenylalanine. The abundances of AAA were also higher in RK_*BA* samples than in RK_*AO* samples (Figure 3).

### 3.3. Amino Acids

Amino acids serve as the N source for fermenter microorganisms, and the composition and content in the fermented product vary depending on the activity of microbial proteolytic and biosynthetic enzymes such as protease, aminopeptidase, and aminotransferase [33]. Although protease activity was significantly higher in RK_*BA* samples than in RK_*AO* samples (Figure 4), and the levels of the larger number amino acids were elevated in RK_*BA* than in RK_*AO* (Figure 2), some amino acids were more abundant in RK_*AO* than in RK_*BA*. AAA and BCAA exhibited high relative abundances in RK_*BA*, while five amino acids related to the serine pathway and γ-aminobutyric acid (GABA) showed elevated levels in RK_*AO* (Figure 3). This result is consistent with previous reports that fermented foods produced using *Bacillus* spp. have higher AAA and BCAA content than foods fermented by *Aspergillus* spp. [1,34]. Acetolactate synthase and branched chain aminotransferase catalyze the synthesis of BBCA from pyruvate [35,36], and AAA were synthesized from chorismic acid via 3–6 enzymatic reactions in the shikimic acid pathway [37]. We considered that these pathways and enzymes may be involved in rice *koji* production by *B. amyloliquefaciens*.

In glycine, serine, and threonine metabolism, glycine has a reversible relationship with serine by serine-hydroxymethyltransferase and with threonine by threonine-aldolase, both enzymes that are expressed in microorganisms [38]. Aspartic acid is converted to alanine directly by aspartate 4-decarboxylase and to threonine through four enzymatic reaction [39]. GABA is a bioactive compound in rice that is produced from glutamic acid by glutamate decarboxylase (GAD) [40]. The GAD-encoding gene has been cloned in *A. oryzae*, and GAD purified from *A. oryzae* was shown to have high activity [41]; however, *Bacillus* species lack a GAD-related gene, and as such, have relatively weaker capacities to produce GABA [42].

### 3.4. Lipid Metabolism

Phospholipids composing plant cell membranes are degraded to lysophospholipids and fatty acids by the lipolytic enzyme phospholipase A, which is secreted by the inoculated microbes [43]. Fatty acids were shown to increase with decomposing triacylglycerol in rice *koji* fermentation [44]. LysoPC/PEs represented higher relative contents in RK_*BA*; however, fatty acids were higher in RK_*AO* (Figure 3). Kum *et al.* [4] reported that long-chain fatty acids were increased in the later stages of rice *koji*-*doenjang* fermentation by *Aspergillus* species and were strongly correlated with lipase activity. Although lipolytic enzymes are expressed in both *A. oryzae* and *B. amyloliquefaciens*, the microbial lipid metabolites differed between the rice *koji* prepared with the two organisms.

### 3.5. Phenolic Compounds

Fermentation increases the abundance of bioactive phenolic compounds [5,45]. The TFC values were higher in RK_*BA* than in RK_*AO*, whereas TPC was slightly more abundant in RK_*AO* than in RK_*BA* (Figure 4B,C). Metabolite profiling with the correlation assay confirmed that the detected metabolites contributed to these total contents. With this assay, we detected phenolic acids such as ferulic acid and 4-hydroxybenzoic acid, as well as flavonoids such as two apigenin glycosides, two chrysoeriol glycosides, two tricin glycosides, and tricin.

Phenolic acids such as ferulic acid exist in the bound form in cell wall polysaccharides and are released by feruloyl esterase [46]. Although feruloyl esterase is a common microbial enzyme, it is most commonly secreted by fungi rather than bacteria [47]. Glycosidic flavonoids in cell vacuoles are subjected to reactions such as glycosylation, de-glycosylation, methylation, glucuronidation, and sulfate conjugation [48]. Flavonoid glycosylation is accomplished by glycosyltransferases, whose genes are expressed in *B. cereus*, *B. licheniformis, Streptomyces*, and *Xanthomonas campestris* (e.g., BcGT-1, DSM-13, YjiC, OleD, and XcGT-2). Tricin is considered a deglycosylated metabolite of microbial β-glucosidase from rice *koji.* β-Glucosidase hydrolyzes flavonoid glycosides into their corresponding aglycons [45]. We found that flavonoid aglycons and glycosides increased with fermentation time, which may have increased the antioxidative effects (Figure 4A). Flavonoids, particularly aglycon, are potential antioxidants due to their redox properties [49].

### 3.6. Siderophores

Siderophores are iron-chelating compounds secreted by microbes. Bacillibactin is a siderophore secreted by *B. amyloliquefaciens* to acquire iron [50]. We observed bacillibactin in RK_*BA* samples but not in any other samples. The *dhb* gene clusters of *Bacillus* species encode multi-enzyme metabolic networks that include isochorismatase, isochorismatase synthase, and 2,3-dihydroxybenzoate-AMP ligase; these networks produce bacillibactin derived from chorismic acid [51]. Kojic acid, which is produced from glucose by *Aspergillus* species, also contains a siderophore structure [5,52]. Relative contents of kojic acid were higher in RK_*AO* than in RK_*BA*.

## 4. Materials and Methods

### 4.1. Chemicals and Reagents

Water, methanol, and acetonitrile were purchased from Fisher Scientific (Pittsburgh, PA, USA). Dipotassium hydrogen phosphate, potassium dihydrogen phosphate, sodium chloride, diethylene glycol, and sodium carbonate were purchased from Junsei Chemical Co., Ltd. (Tokyo, Japan). Trichloroacetic acid was purchased from Merck Millipore Co. (Darmstadt, Germany). Methoxyamine hydrochloride, pyridine, *N*-methyl-*N*-(trimethylsilyl)trifluoroacetamide (MSTFA), potassium persulfate, 2,2′-azinobis(3-ethylbenzothiazoline-6-sulfonic acid) diammonium salt (ABTS), Folin-Ciocalteu’s phenol, soluble starch, potassium sodium tartrate tetrahydrate, 3,5-dinitrosalicylic acid, sodium hydroxide, acetic acid, sodium acetate, and the standards 6-hydroxy-2,5,7,8-tetramethylchroman-2-carboxylic acid (Trolox), gallic acid, naringin, maltose, tyrosine, and *p*-nitro-phenol were obtained from Sigma-Aldrich (St. Louis, MO, USA).

### 4.2. Inoculum and Rice Koji Fermentation

*A. oryzae* KCCM 11300P and *B. amyloliquefaciens* KCCM 11718P were used for fermentation of rice. To make rice *koji*, 1 kg of rice was submerged in water for 30 min, and the water was drained off. The soaked rice was sterilized for 15 min using an autoclave. Steamed rice (50 g) was inoculated with the fungal strain *A. oryzae*, and incubated at 35 °C for 5 days. Then, the cultured rice was mixed with steam rice again (0.2%, *w*/*w*), and fermented at 37 °C for 36 h. The bacterial strain *B. amyloliquefaciens* was grown in 200 mL of nutrient broth (pH 7.0) at 37 °C with shaking at 200 rpm for 24 h in a 500 mL flask. The cultured broth was also mixed with steamed rice (2.0%, *v*/*w*) and fermented at 37 °C for 36 h. Fermented samples were obtained at 12 h intervals and stored at −20 °C before analysis.

### 4.3. Sample Preparation for Metabolite Profiling

The rice *koji* was dried using a freeze dryer and ground using a mortar. Each sample powder (3 g) was extracted with 30 mL of 80% aqueous methanol by sonication for 10 min, and then shaken at 200 rpm for 24 h. Next, the sample mixtures were centrifuged at 5000 rpm for 10 min at 4 °C. The supernatants were filtered using Millex^®^GP 0.22 μm filters (Merck Millipore, Billerica, MA, USA) and dried in a speed vacuum concentrator (Biotron, Seoul, Korea). The extraction yield from each sample was calculated. For the GC-TOF-MS analysis, norvaline was added as an internal standard, and the sample mixture was derivatized. One hundred microliters of methoxyamine hydrochloride (20 mg/mL in pyridine) was added to each dried sample, and the samples were heated at 30 °C for 90 min. Next, 100 μL of the derivatization agent MSTFA was added to each sample, and the derivatization reactions were heated at 37 °C for 30 min. For UHPLC-LTQ-IT-MS/MS, formononetin was added as an internal standard. The dried samples were dissolved in 200 μL of 80% aqueous methanol, and filtered using 0.2 μm polytetrafluoroethylene (PTFE) filters.

### 4.4. GC-TOF-MS Analysis

The gas chromatography time-of-flight mass spectrometry analysis was performed using an Agilent 7890 A gas chromatograph, a Pegasus HT TOF-MS (Leco Corporation, St. Joseph, MI, USA), and an Agilent 7693 autosampler (Agilent, Atlanta, GA, USA). We used an RTx-5MS GC column (30 m length × 0.25 mm i.d. × 0.25 μm film thickness, J & W Scientific, Folsom, CA, USA), with the carrier gas helium at a constant flow rate of 1.5 mL/min. One microliter of each derivatized sample was injected in split mode (5:1). The temperatures of the injector and ion source were 250 °C and 230 °C, respectively. The column temperature was held constant at 75 °C for 2 min, subsequently increased to 300 °C at a rate of 15 °C/min, and finally held constant for 3 min. The MS data acquisition rate was 10 scans/s, with an *m*/*z* range of 50–1000. For the GC-TOF-MS analysis, three replicates of each sample were tested.

### 4.5. UHPLC-LTQ-IT-MS/MS Analysis

Ultrahigh-performance liquid chromatography linear trap quadrupole ion trap tandem mass spectrometry was performed using an LTQ ion trap mass spectrometer equipped with a binary solvent delivery system, RS autosampler, electrospray interface (Thermo Fisher Scientific, San José, CA, USA), DIONEX UltiMate 3000 RS Pump, RS Column Compartment, and RS Diode Array Detector (Dionex Corporation, Sunnyvale, CA, USA). Each injected sample (10 μL) was separated on a Syncronis C18 column (100 mm × 2.1 mm, 1.7 μm particle size; Thermo Scientific) at a flow rate of 0.3 mL/min. The mobile phases consisted of 0.1% formic acid in water (*v*/*v*) (Solution A) and 0.1% formic acid in acetonitrile (*v*/*v*) (Solution B). The solvent gradient program began with 10% Solution B/90% Solution A for 1 min, followed by a 14-min constant-rate increase to 100% Solution B, followed by 3 min of 100% Solution B, followed by a 1 min constant-rate decrease to 10% Solution B/90% Solution A, and finally 3 min of 10% Solution B/90% Solution A. The total run time was 22 min. The photodiode array detection range was 200–600 nm. Electron spray ionization was performed in the positive and negative ion modes within an *m*/*z* range of 100–1000. Other instrument parameters were as follows: capillary temperature, 275 °C; source voltage, ±5 kV; and capillary voltage, 39 V. Triplicate UHPLC-LTQ-IT-MS/MS runs were performed for each sample.

### 4.6. Data Processing and Multivariate Statistical Analysis

The GC-TOF-MS and UHPLC-LTQ-IT-MS/MS data were converted to netCDF (*.cdf) format using Leco ChromaTOF and Thermo Xcalibur software. The metAlign software package [53] was used to align the netCDF data. After alignment, the resulting peak list was exported to a Microsoft Excel file (.xls) that included the corrected peak retention times (min), mass to charge ratios (*m*/*z*), and peak areas. The peak area values were converted according to the extraction yield of each sample and log_10_ transformed in Excel. Multivariate statistical analyses were performed using SIMCA-P+ 12.0 software (Umetrics, Umea, Sweden) to compare metabolite differences between rice *koji* fermented with the fungus and rice *koji* fermented with the bacterium. We performed a principal component analysis (PCA), partial least-squares discriminant analysis (PLS-DA), and orthogonal partial least square discriminant analysis (OPLS-DA). The data sets were auto-scaled (unit variance scaling) and mean-centered in a column-wise fashion. Metabolites with VIP values greater than 0.7 in PLS1 or PLS2 and *p*-values less than 0.05 were selected for further analysis.

### 4.7. Determination of Antioxidant Activity and Total Phenolic and Flavonoid Content

To determine the antioxidant activity of samples, we used an ABTS assay, a total phenolic content (TPC) assay, and a total flavonoid content (TFC) assay. All experiments were performed in triplicate.

The ABTS assay was conducted using a method modified from Re *et al.* [54]. A stock solution was prepared by dissolving 7 mM ABTS in 2.45 mM potassium persulfate solution, incubating the solution in a water bath at 60 °C for 20 min, and storing this solution for 12 h at room temperature. After the last step, this stock solution was dark blue in color. For analysis, the solution was diluted in distilled water to an absorbance of 0.7 ± 0.02 at 750 nm. We used a spectrophotometer to measure absorbance (Spectronic Genesys 6, Thermo Electron, Madison, WI, USA). Each sample (20 μL) was added along with stock ABTS (180 μL) into a well of a 96-well plate. The plate was incubated at room temperature for 6 min in the dark, and the absorbance of each well was measured at 750 nm. Trolox was used as a standard, and the results are presented as the Trolox equivalent antioxidant capacity (TEAC) concentration (mM) per milligram of *koji*. The standard curves ranged from 0.0156 mM to 0.5 mM.

For the TFC assay, we followed a method outlined by Davis [55] with slight modifications. Twenty microliters of each sample, 20 μL of 1 N NaOH, and 180 μL of 90% diethylene glycol were mixed in a 96-well plate. The mixture was incubated for 60 min at room temperature, and the absorbance was measured at 405 nm using the spectrophotometer. TFC is expressed as the naringin equivalent (NE) concentration (ppm) per milligram of *koji*. The standard concentration curve ranged from 1.56 to 200 ppm.

The TPC determination followed a method used by Yildirim *et al.* [56] with slight modifications. Briefly, 20 μL of each sample and 100 μL of 0.2 N Folin-Ciocalteu reagent were added to a 96-well plate and incubated at room temperature for 6 min. Next, 80 μL of 7.5% sodium carbonate (Na_2_CO_3_) solution was added to the mixture, reacted for 60 min at room temperature, and evaluated at 750 nm. The results are presented as gallic acid equivalent (GE) concentrations (ppm) per milligram of *koji* in a standard concentration range of 3.91–500 ppm.

### 4.8. Determination of Enzymatic Activities

Enzyme activity assays were conducted for α-amylase, protease, and β-glycosidase. To determine enzyme activity, each rice *koji* sample (10 g) was extracted with 90 mL of distilled water by shaking at 120 rpm and 30 °C for 1 h. The mixture was centrifuged at 5000 rpm and 4 °C for 5 min. Next, the supernatants were filtered using 0.2 μm PTFE filters.

To assess α-amylase activity, 1 mL of 1% soluble starch solution in 20 mM sodium phosphate buffer with 6.7 mM sodium chloride (pH 6.9) was added to the extracted enzyme solution. The mixture was incubated at 55 °C for 10 min. Next, each sample was mixed with a color reagent solution (96 mM 3,5-dinitrosalicylic acid solution added to sodium potassium tartrate solution) and boiled at 100 °C for 15 min. The heated samples were cooled on ice and diluted with 9 mL of distilled water. The absorbance of each sample was read at 540 nm. One unit of α-amylase activity was defined as the quantity of α-amylase that induced a change of 1 mg of maltose in a 1% soluble starch solution in 1 min [8].

Protease activity was assayed using a modification on the method described by Kum *et al.* [4]. One milliliter of the extracted enzyme solution was mixed with 5 mL of 0.6% casein solution, dissolved in 0.1 M phosphate buffer (pH 7), and reacted at 37 °C for 10 min. After 10 min, the reaction was stopped by adding 5 mL of 0.4 M trichloroacetic acid and incubating at 37 °C for 30 min. The precipitate was filtered with a 0.2 μm PTFE filter. Next, 5 mL of 0.4 M sodium carbonate and 1 mL of thrice-diluted 2 N folin reagent were added to 2 mL of filtrate. The mixture was incubated at 37 °C for 30 min, and its absorbance measured at 660 nm. One unit of protease activity was defined as the amount of protease required to release 1 μg of tyrosine per minute from 0.6% casein under corresponding conditions.

The β-glucosidase activity assay followed the method of Zhang *et al.* [20], with slight modifications. Nine millimolar *p-*nitrophenol β-d-glucopyranoside (*p-*NPG) in 0.1 M sodium acetate buffer (pH 4.6) was prepared as a substrate solution. One milliliter of extracted enzyme sample was added to 1 mL of substrate solution and 8 mL of sodium acetate buffer, and then reacted at 37 °C for 30 min. After 30 min, 10 mL of 0.4 M sodium carbonate was added to stop the reaction, and the absorbance was measured at 400 nm. One unit of β-glucosidase activity refers to the quantity of **β**-glucosidase needed to liberate 1 nmol *p*-nitrophenol from *p-*NPG in 1 min under the specified conditions. All experiments were performed in triplicate.

## 5. Conclusions

In conclusion, we investigated the fermentative behavior of *A. oryzae* (a fungus) and *B. amyloliquefaciens* (a bacterium) over different fermentation times. The rice *koji* samples exhibited distinct metabolites and enzymatic activities based on fermentation duration and fermenter species. The RK_*AO* groups had relatively high contents of carbohydrate metabolism intermediates such as sugars and sugar alcohols, organic acids, and phenolic acids and lipid metabolism intermediate, fatty acids. The RK_*BA* groups had relatively high abundances of flavonoids, lysophospholipids, and amino acids, especially AAA and BCAA. The existence and expression levels of certain genes affected the metabolisms in these two microorganisms. The heightened flavonoid content of RK_*BA* led to higher antioxidant activity in these samples relative to that of RK_*AO*. These metabolic, enzymatic, and bioactivity characteristics of rice *koji* may be used to optimize choices of fermenter species and fermentation durations to enhance the quality of fermented food.

## Supplementary Materials

The following are available online at http://www.mdpi.com/1420-3049/21/6/773/s1, Figure S1: Partial least square-discriminate analysis (PLS-DA) score plot for rice *koji* fermented with *A. oryzae* (RK_*AO*) or *B. amlyoliquefaciens* (RK_*BA*) during fermentation times obtained from GC-TOF-MS (a) and UHPLC-LTQ-IT-MS/MS (b), Figure S2: Comparison of pH and total acidity of rice *koji* fermented with *A. oryzae* (RK_*AO*, closed circle) or *B. amlyoliquefaciens* (RK_*BA*, open circle) during fermentation times, Table S1: Discriminative metabolites and their relative contents in rice *koji* fermented with *A. oryzae* (RK_*AO*) or *B. amyloliquefaciens* (RK_*BA*) during fermentation using GC-TOF-MS, Table S2: Discriminative metabolites and their relative contents in rice *koji* fermented with *A. oryzae* (RK_*AO*) or *B. amyloliquefaciens* (RK_*BA*) during fermentation using UHPLC-LTQ-IT-MS/MS.
