# Supplementary material for: Metabolomic Profiles of Aspergillus oryzae and Bacillus amyloliquefaciens During Rice Koji Fermentation
Source: Molecules. 2016 Jun 14;21(6):773. doi: 10.3390/molecules21060773 (PMC6273993; doi:10.3390/molecules21060773)
Supplement: Supplementary file 1 [file molecules-21-00773-s001.pdf]

Supplementary Materials: Metabolomic Profiles of *Aspergillus oryzae* and *Bacillus amyloliquefaciens* During Rice *koji* Fermentation

Da Eun Lee Sunmin Lee, Eun Seok Jang, Hye Won Shin, Byoung Seok Moon and Choong Hwan Lee

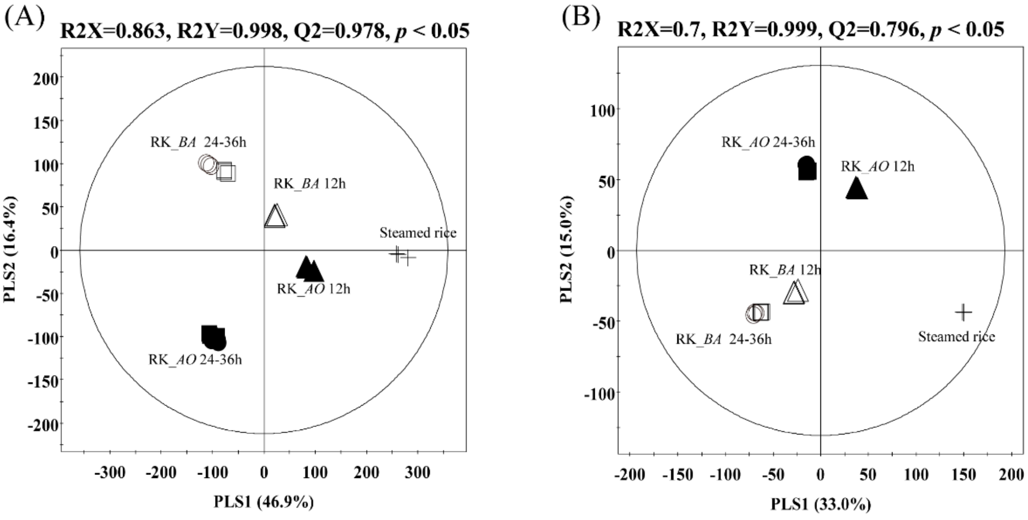

Figure S1. Partial least square-discriminate analysis (PLS-DA) score plot for rice *koji* fermented with *A. oryzae* (*RK_AO*) or *B. amlyoliquefaciens* (RK_*BA)* during fermentation times obtained from GC-TOF-MS (**a**) and UHPLC-LTQ-IT-MS/MS (**b**). (+, Steamed rice; open symbol, RK_*AO*; closed symbol, RK_*BA*; △, ▲, 12 h; □, ■, 24 h; ○, ●, 36 h).

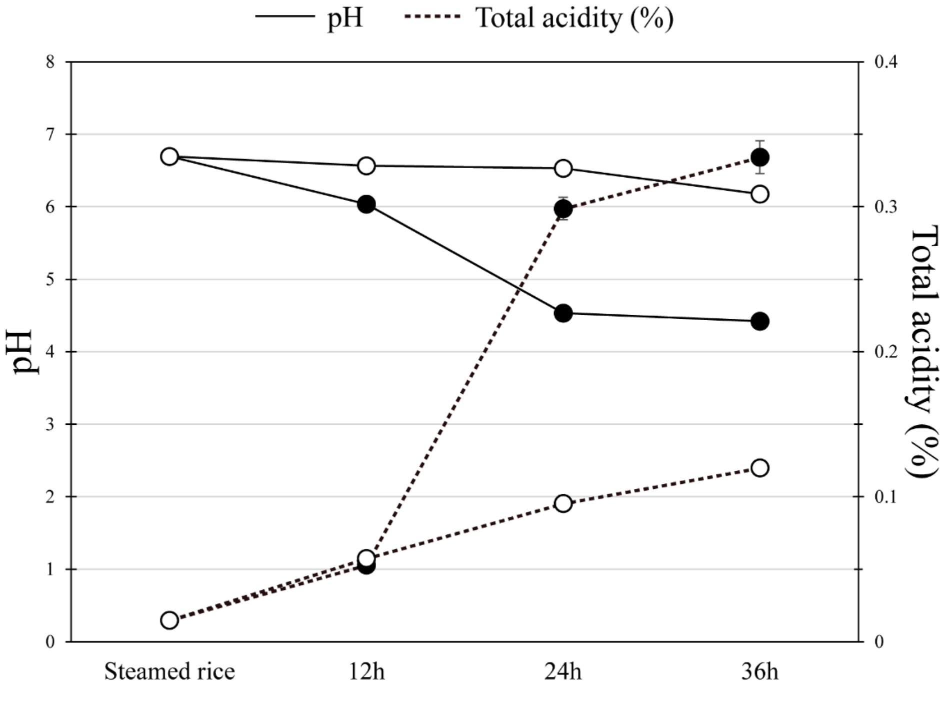

Figure S2. Comparison of pH and total acidity of rice *koji* fermented with *A. oryzae* (RK_*AO*, closed circle) or *B. amlyoliquefaciens* (RK_*BA*, open circle) during fermentation times.

Table S1. Discriminative metabolites and their relative contents in rice *koji* fermented with *A. oryzae* (RK_*AO*) or *B. amyloliquefaciens* (RK_*BA*) during fermentation using GC-TOF-MS.

Peak No.	Tentative Identification [a,b]	RT (min) [c]	Identified Ion (*m*/*z*) [d]	TMS [e]	Relative Contents (Peak Area [log_{10}]) [f]						
					Steamed rice	RK_*AO* 12 h	RK_*AO* 24 h	RK_*AO* 36 h	RK_*BA* 12 h	RK_*BA* 24 h	RK_*BA* 36 h
	**Sugars and sugar alcohols**										
1	Glycerol	7.13	117	3	3.90 ± 0.04	6.09 ± 0.00	7.01 ± 0.01	7.10 ± 0.05	6.32 ± 0.04	5.60 ±0.04	5.60 ± 0.04
2	Erythritol	9.28	117	4	3.95 ± 0.05	3.8 ± 0.05	5.51 ± 0.03	5.93 ± 0.02	4.61 ± 0.12	4.26 ±0.05	4.26 ± 0.05
3	Pentitol	10.2	231	4	3.77 ± 1.02	4.85 ± 0.39	4.29 ± 0.06	3.87 ± 0.06	5.33 ± 0.17	5.71 ±0.11	5.71 ± 0.11
4	Xylose	10.5	103	4	3.86 ± 0.06	4.16 ± 0.05	5.30 ± 0.05	5.42 ± 0.03	5.16 ± 0.12	4.62 ±0.06	4.62 ± 0.06
5	Xylitol	10.89	217	5	4.66 ± 0.05	4.22 ± 0.07	5.01 ± 0.00	6.31 ± 0.03	4.26 ± 0.08	3.98 ±0.04	3.98 ± 0.04
6	Fructose	12.15	103	5	5.52 ± 0.01	5.65 ± 0.03	5.84 ± 0.07	5.77 ± 0.07	5.68 ± 0.09	4.97 ±0.08	4.97 ± 0.08
7	Glucose	12.28	319	5	4.94 ± 0.01	5.82 ± 0.02	6.11 ± 0.07	6.04± 0.02	5.83 ± 0.06	5.84 ±0.02	5.84 ± 0.02
8	Sorbitol	12.53	421	6	4.00 ± 0.01	4.27 ± 0.02	4.74 ± 0.00	4.50 ± 0.01	0.51 ± 0.89	0.60 ±1.04	0.60 ± 1.04
9	myo-Inositol	13.53	305	6	4.53 ± 0.01	4.96 ± 0.01	5.66 ± 0.00	5.58 ± 0.01	4.40 ± 0.06	3.89 ±0.05	3.89 ± 0.05
10	Maltose	17.13	204	8	4.85 ± 0.12	6.52 ± 0.04	6.11 ± 0.06	6.05 ± 0.08	6.19 ± 0.01	6.54 ±0.01	6.54 ± 0.01
	**Organic acids**										
11	Lactic acid	4.93	117	2	4.69 ± 0.05	4.76 ± 0.04	4.86 ± 0.07	5.05± 0.07	4.98 ± 0.06	5.42 ±0.07	5.42 ± 0.07
12	Oxalic acid	5.68	133	2	1.97 ± 0.01	2.72 ± 0.03	3.20 ± 0.05	3.12 ± 0.02	2.93 ± 0.06	3.33 ±0.05	3.33 ± 0.05
13	Malonic acid	6.44	233	2	2.55 ± 0.08	1.87 ± 0.03	2.17 ± 0.11	0.70 ± 1.22	2.52 ± 0.10	2.85 ±0.11	2.85 ± 0.11
14	Succinic acid	7.49	247	2	3.75 ± 0.13	3.79 ± 0.02	4.86 ± 0.02	4.56 ± 0.04	3.79 ± 0.06	4.49 ±0.05	4.49 ± 0.05
15	Glyceric acid	7.69	189	3	4.19 ± 0.11	3.79 ± 0.03	4.61 ± 0.08	4.59 ± 0.09	3.53 ± 0.11	3.87 ±0.06	3.87 ± 0.06
16	Fumaric acid	7.79	245	2	3.92 ± 0.10	3.68 ± 0.04	4.68 ± 0.02	4.46 ± 0.03	3.67 ± 0.08	3.93 ±0.12	3.93 ± 0.12
17	Malic acid	9.09	233	3	4.29 ± 0.08	4.07 ± 0.03	4.95 ± 0.04	4.64 ± 0.05	3.38 ± 0.06	3.76 ±0.04	3.76 ± 0.04
18	Kojic aicd	10.6	271	2	1.46 ± 0.08	3.23 ± 0.08	3.31 ± 0.09	4.39 ± 0.05	3.18 ± 0.07	2.39 ±0.41	2.39 ± 0.41
19	Shikimic acid	11.54	204	4	3.52 ± 0.08	3.11 ± 0.21	3.56 ± 0.18	3.17 ± 0.17	3.57 ± 0.12	3.90 ±0.12	3.90 ± 0.12
20	Citric acid	11.67	273	4	4.21 ± 0.10	4.73 ± 0.04	6.55 ± 0.01	6.56 ± 0.01	5.11 ± 0.07	4.05 ±0.07	4.05 ± 0.07
21	Gluconic acid	12.97	292	5	4.73 ± 0.02	4.95 ± 0.00	5.20 ± 0.01	5.10 ± 0.01	4.67 ± 0.06	4.25 ±0.04	4.25 ± 0.04
	**Phenolic acids**										
22	4-Hydroxybenzoic acid	10.2	267	2	3.25 ± 0.10	3.01± 0.02	4.17 ± 0.07	3.76 ± 0.06	3.02± 0.11	1.80 ±1.56	1.80 ± 1.56
23	Ferulic acid	13.42	249	2	2.90 ± 0.04	3.51 ± 0.02	3.87 ± 0.02	3.38 ± 0.03	2.61 ± 0.17	2.08 ±0.22	2.08 ± 0.22
	**Amino acids**										
24	Alanine	5.37	116	2	4.49 ± 0.07	4.22 ± 0.03	5.41 ± 0.06	5.26 ± 0.07	4.22 ± 0.16	4.95 ±0.09	4.95 ± 0.09
25	Valine	6.57	144	2	3.75 ± 0.03	4.19 ± 0.06	5.67 ± 0.05	5.52 ± 0.05	5.21 ± 0.02	6.09 ±0.05	6.09 ± 0.05
26	Leucine	7.12	158	2	3.53 ± 0.03	4.65 ± 0.02	5.65 ± 0.11	4.91 ± 0.01	5.22 ± 0.05	6.10 ±0.03	6.10 ± 0.03
27	Isoleucine	7.34	158	2	3.37 ± 0.03	4.07± 0.02	5.58 ± 0.03	5.60 ± 0.03	4.92 ± 0.04	5.84 ±0.04	5.84 ± 0.04

28	Proline	7.39	142	2	4.48 ± 0.06	4.98 ± 0.01	5.72 ± 0.06	5.69 ± 0.02	4.21 ± 0.03	5.14 ±0.05	5.14 ± 0.05
29	Glycine	7.47	174	3	4.96 ± 0.02	4.73 ± 0.01	5.83 ± 0.02	4.93 ± 0.03	4.62 ± 0.05	5.26 ±0.06	5.26 ± 0.06
30	Serine	7.87	204	3	4.34 ± 0.03	4.39 ± 0.05	5.59 ± 0.03	5.49 ± 0.03	4.38 ± 0.04	4.70 ±0.08	4.70 ± 0.08
31	Threonine	8.22	219	3	3.52 ± 0.06	3.71 ± 0.08	5.14 ± 0.05	5.08± 0.04	3.96 ± 0.04	4.51 ±0.06	4.51 ± 0.06
32	Methionine	9.36	176	2	2.66 ± 0.12	3.39 ± 0.07	5.06± 0.04	4.87 ± 0.04	4.45 ± 0.05	5.15 ±0.07	5.15 ± 0.07
33	Aspartic acid	9.36	232	3	4.42 ± 0.06	4.23 ± 0.05	5.39 ± 0.05	5.35 ± 0.05	4.10 ± 0.03	4.15 ±0.08	4.15 ± 0.08
34	Pyroglutamic acid	9.41	156	2	5.17 ± 0.06	4.95 ± 0.07	6.02± 0.06	5.87 ± 0.05	5.36 ± 0.04	5.87 ±0.08	5.87 ± 0.08
35	GABA	9.43	174	3	5.04± 0.02	3.83 ± 0.04	6.06± 0.02	6.01± 0.03	4.62 ± 0.09	4.54 ±0.02	4.54 ± 0.02
36	Glutamic acid	10.14	246	3	4.33 ± 0.06	4.04± 0.01	5.16 ± 0.02	4.89 ± 0.01	4.70 ± 0.10	5.28 ±0.02	5.28 ± 0.02
37	Phenylalanine	10.24	218	2	3.19 ± 0.17	3.98 ± 0.11	5.42 ± 0.06	5.22 ± 0.06	5.11 ± 0.10	5.89 ±0.05	5.89 ± 0.05
38	Ornithine	11.63	142	4	2.30 ± 0.06	2.93 ± 0.03	3.91 ± 0.08	3.96 ± 0.08	3.69 ± 0.07	4.56 ±0.06	4.56 ± 0.06
39	Lysine	12.35	156	4	3.48 ± 0.09	3.29 ± 0.12	3.22 ± 0.09	ND [g]	4.38 ± 0.12	4.93 ±0.06	4.93 ± 0.06
40	Tyrosine	12.48	218	3	3.96 ± 0.06	3.71 ± 0.06	3.97 ± 0.02	4.11 ± 0.02	5.35 ± 0.08	6.05±0.04	6.05± 0.04
41	Tryptophan	14.27	202	3	3.71 ± 0.11	3.57 ± 0.10	4.78 ± 0.13	4.66 ± 0.20	4.21 ± 0.21	4.93 ±0.12	4.93 ± 0.12
					**Fatty acids**						
42	Palmitic acid	13.05	132	1	5.10 ± 0.01	5.13 ± 0.01	5.68 ± 0.01	5.59 ± 0.01	5.48 ± 0.08	5.31 ±0.03	5.31 ± 0.03
43	Linoleic acid	14.09	262	1	4.69 ± 0.00	4.65 ± 0.01	4.98 ± 0.01	4.93 ± 0.01	4.92 ± 0.03	4.69 ±0.02	4.69 ± 0.02
44	Oleic acid	14.12	339	1	4.22 ± 0.01	4.47 ± 0.00	4.77 ± 0.00	4.73 ± 0.02	4.79 ± 0.03	4.54 ±0.02	4.54 ± 0.02
45	Linolenic acid	14.16	335	1	3.28 ± 0.03	3.09± 0.16	3.72 ± 0.18	3.59 ± 0.17	3.45 ± 0.17	3.47 ±0.11	3.47 ± 0.11
46	Strearic acid	14.23	341	1	3.25 ± 0.04	3.3 ± 0.01	3.84 ± 0.02	3.71 ± 0.03	3.58 ± 0.07	3.71 ±0.03	3.71 ± 0.03
					**Vitamins**						
47	Nicotinic acid	7.36	180	1	4.3 ± 0.09	3.6 ± 0.05	3.73 ± 0.05	4.00 ± 0.03	1.18 ± 1.02	0.85 ±0.85	0.85 ± 0.85

[a] Tentative metabolites identified by mass spectrum consistent with those of standard compound, NIST, and in-house library; [b] The different metabolites based on variable importance projection (VIP) analysis with a cutoff value of 0.7 and a p value <0.05; [c] Retention time; [d] The selected ion is m/z value for identification and quantification; [e] TMS: trimethylsilyl; [f] The relative contents of metabolites were represented as peak area transformed by $\log_{10}$. Mean (n = 3) ± standard deviation; [g] ND: Not detected.

Table S2. Discriminative metabolites and their relative contents in rice *koji* fermented with *A. oryzae* (RK_*AO*) or *B. amyloliquefaciens* (RK_*BA*) during fermentation using UHPLC-LTQ-IT-MS/MS.

Peak No.	Tentative Identification [a, b]	RT (min) [c]	Measured Mass $[M + H]^+$	MS^n Fragment Ions	UV (nm)	Relative Contents (Peak Area [log_{10}]) [d]						
						Steamed Rice	RK_*AO* 12 h	RK_*AO* 24 h	RK_*AO* 36 h	RK_*BA* 12 h	RK_*BA* 24 h	RK_*BA* 36 h
						**Flavonoids**						
48	Apigenin-C-glucosyl-C-arabinoside	6.7	565	565 > 547 > 499	272, 334	3.72 ± 0.01	ND [e]	ND	ND	4.81 ± 0.01	4.91 ± 0.01	4.93 ± 0.01
49	Isovitexin-O-glucoside	7.1	595	595 > 577 > 529	309	3.30 ± 0.03	ND	ND	ND	4.51 ± 0.04	4.71 ± 0.03	4.71 ± 0.01
50	Chrysoeriol-hexoside	7.8	463	-	322	2.98 ± 0.02	ND	ND	ND	4.37 ± 0.01	4.56 ± 0.02	4.61 ± 0.01
51	Chrysoeriol-rutinoside	8.1	609	-	246, 322	3.30 ± 0.02	ND	ND	ND	4.08± 0.04	4.28 ± 0.03	4.40 ± 0.03
52	Tricin-7-O-rutinoside	8.1	639	639 > 493 > 331	340	ND	ND	ND	ND	4.52 ± 0.01	4.65 ± 0.02	4.67 ± 0.01
53	Tricin-O-glucoside	8.5	493	493 > 331	330	3.00± 0.04	ND	ND	ND	4.11 ± 0.04	4.27 ± 0.02	4.27 ± 0.02
54	Tricin	10.8	331	-	-	3.98 ± 0.02	4.31 ± 0.01	4.53 ± 0.01	4.45 ± 0.03	4.52 ± 0.02	4.59 ± 0.02	4.56 ± 0.02
						**Fatty acids**						
55	Pinellic acid	10.9	353 *	-	217, 302	4.37 ± 0.01	5.00± 0.02	5.34 ± 0.01	5.34 ± 0.04	5.03 ± 0.03	5.23 ± 0.04	5.26 ± 0.00
56	Hydroxy-oxo-octadecenoic acid	13.1	313	313 > 297 > 279	221, 273	4.78 ± 0.03	4.74 ± 0.02	4.98 ± 0.01	4.98 ± 0.02	4.64 ± 0.04	4.68 ± 0.05	4.69 ± 0.06
						**Lysophospholipids**						
57	LysoPE14:0	13.5	426	426 > 408 > 365	220	4.63 ± 0.15	4.20 ± 0.02	4.76 ± 0.02	4.90 ± 0.02	5.00 ± 0.05	5.40 ± 0.03	5.44 ± 0.01
58	LysoPC14:0	13.7	468	468 > 450	220	5.85 ± 0.03	5.87 ± 0.77	6.41 ± 0.11	6.45 ± 0.04	6.42 ± 0.05	6.63 ± 0.04	6.62 ± 0.09
59	LysoPC18:3	13.9	518	519 > 500	221	5.27 ± 0.08	4.83 ± 0.27	5.43 ± 0.06	5.11 ± 0.51	5.52 ± 0.09	5.74 ± 0.02	5.78 ± 0.03
60	LysoPC16:1	14.3	494	494 > 476	220	4.67 ± 0.08	4.19 ± 0.21	4.80 ± 0.07	4.71 ± 0.09	4.86 ± 0.08	4.89 ± 0.10	4.98 ± 0.03
61	LysoPE18:2	14.5	478	478 > 460 > 263	-	5.26 ± 0.03	5.09± 0.05	5.67 ± 0.02	5.80 ± 0.02	5.83 ± 0.04	6.30 ± 0.03	6.40 ± 0.01
62	LysoPC18:2	14.9	520	520 > 502	301	5.74 ± 0.15	5.97 ± 0.20	6.40 ± 0.13	6.38 ± 0.07	6.27 ± 0.06	6.41 ± 0.06	6.50 ± 0.04
63	LysoPE16:0	15.2	454	454 > 436 > 393	223	5.21 ± 0.02	4.96 ± 0.08	5.56 ± 0.01	5.64 ± 0.03	5.58 ± 0.04	6.03± 0.05	6.10 ± 0.02
64	LysoPC16:0	15.7	496	496 > 478, 184	224	5.75 ± 0.17	6.22 ± 0.10	6.56 ± 0.07	6.39 ± 0.10	6.41 ± 0.08	6.59 ± 0.15	6.57 ± 0.10
65	LysoPC18:1	16.3	522	522 > 504	223, 277	5.00± 0.16	5.28 ± 0.06	5.40 ± 0.65	5.41 ± 0.20	5.60 ± 0.17	5.69 ± 0.11	5.79 ± 0.13
						**Siderophores**						
66	Bacillibactin	9.5	883	883 > 690 > 672	209, 247, 313	ND	ND	ND	ND	5.16 ± 0.04	5.91 ± 0.02	6.16 ± 0.02
						**Unknown**						
67	N.I 1	1.5	294	294 > 276 > 258	225, 261, 364	4.57 ± 0.02	6.32 ± 0.02	6.91 ± 0.01	6.81 ± 0.01	5.65 ± 0.01	5.44 ± 0.01	5.53 ± 0.01
68	N.I 2	7.0	480	480 > 462 > 396	270	ND	4.56 ± 0.02	5.78 ± 0.01	5.30 ± 0.01	ND	ND	ND
69	N.I 3	9.4	420	420 > 402 > 384	-	ND	ND	ND	ND	5.35 ± 0.03	5.65 ± 0.04	5.70 ± 0.03
70	N.I 4	10.8	333	333 > 297 > 279	214, 344	4.39 ± 0.03	4.64 ± 0.03	4.87 ± 0.02	4.81 ± 0.03	4.40 ± 0.03	4.28 ± 0.05	4.34 ± 0.02
71	N.I 5	11.7	402	402 > 384 > 366	218, 300	ND	ND	ND	ND	5.75 ± 0.05	5.96 ± 0.04	6.00 ± 0.03
72	N.I 6	13.0	588	588 > 570 > 552	221, 272	5.08± 0.03	5.25 ± 0.01	5.34 ± 0.02	5.43 ± 0.02	5.02 ± 0.04	4.29 ± 0.04	ND
73	N.I 7	13.2	638	638 > 620 > 602	221, 271	4.70 ± 0.03	4.86 ± 0.01	4.97 ± 0.01	5.07 ± 0.01	4.62 ± 0.03	ND	ND
74	N.I 8	14.0	640	640 > 622 > 604	366	5.64 ± 0.01	6.16 ± 0.03	6.37 ± 0.03	6.07± 0.67	6.02± 0.04	5.26 ± 0.02	5.20± 0.09

[a] Tentative metabolites identified by comparing data to published literature and an in-house library; [b] The different metabolites based on variable importance projection(VIP) analysis with a cutoff value of 0.7and a *p*-value <0.05; [c] Retention time; [d] The relative contents of metabolites were represented as peak area transformed by log_{10}. Mean (n = 3) ± standard deviation; [e] ND: Not detected; * Adduct ion is sodium, $[M + Na]^+$.
